# Osteosclerotic change as a therapeutic response to gefitinib in symptomatic non-small cell lung cancer bone metastasis

**DOI:** 10.1186/s12890-022-02226-1

**Published:** 2022-12-29

**Authors:** Michihito Miyagi, Hirohisa Katagiri, Hideki Murata, Junji Wasa, Toshiaki Takahashi, Haruyasu Murakami, Hideyuki Harada, Keita Mori, Mitsuru Takahashi

**Affiliations:** 1 Devision of Orthopedic Oncology, Shizuoka Cancer Center, Shizuoka, Japan; 2 Devision of Thoracic Oncology, Shizuoka Cancer Center, Shizuoka, Japan; 3 Division of Radiation Therapy, Radiation and Proton Therapy Center, Shizuoka Cancer Center, Shizuoka, Japan; 4 Department of Biostatistics, Clinical Research Center, Shizuoka Cancer Center, Shizuoka, Japan

**Keywords:** Bone metastases, Non-small cell lung cancer (NSCLC), Osteosclerosis, Epidermal growth factor receptor tyrosine kinase inhibitor (EGFR-TKI)

## Abstract

**Background:**

Despite improvement in the overall survival of patients with non-small cell lung cancer (NSCLC) harboring epidermal growth factor receptor (EGFR) mutation, the effects of EGFR tyrosine kinase inhibitor (EGFR-TKI) treatment on bone metastasis remain unclear. This study investigated radiological responses to gefitinib regarding bone metastasis in patients.

**Methods:**

We treated 260 patients with NSCLC and symptomatic bone metastasis. Thirty-seven patients harboring EGFR mutation were treated with gefitinib for more than 30 days and followed up for more than 3 months (GEF group). We performed a retrospective observational study by selecting 36 cases without EGFR-TKI treatment, at least 3 months of follow-up, and at least two radiological evaluations as the control group. We assessed the best overall radiological response, interval from treatment initiation to appearance of a radiological response, and the local response maintenance rate.

**Results:**

The best effect in the GEF group was 98% partial response or better, which was significantly higher than the 57% observed in the control group (*p* < 0.001). The GEF and control groups maintained 83% and 42% local response maintenance rates at one year, respectively (p < 0.001). In the GEF with radiotherapy group, the local response maintenance rate was maintained at 92% at 1 year, while in the GEF without RT group, there was a decrease in the local response maintenance rate from 270 days.

**Conclusion:**

Gefitinib treatment for bone metastases in patients harboring EGFR mutation resulted in a beneficial osteosclerotic change in most patients. Combined gefitinib and radiotherapy provide long-lasting local control of bone metastases.

## Background

Non-small cell lung cancer (NSCLC) accounts for approximately 80–85% of lung cancers and is therefore the most common subtype among lung tumors [1]. According to a study based on autopsy specimens, the proportion of advanced lung cancer patients with bone metastasis was as high as 36% [2]. Furthermore, 30–45% of patients with NSCLC reportedly developed bone metastasis during the course of their illness [3–5]. Many cases of bone metastases from lung cancer are those of osteolytic lesions [6], whereby commonly reported locations include the spine, followed by the rib, pelvis, and femur [4]. The initial symptom of skeletal metastasis is pain; however, without proper treatment for bone metastasis, there is a risk of developing pathological fractures or spinal cord injury, both of which lead to profound debilitation [7].

With conventional cytotoxic chemotherapy, the median overall survival (OS) of patients with NSCLC having bone metastasis was reported to be less than 6 months [8]. However, the life expectancy of patients with advanced NSCLC has improved recently owing to advances in chemotherapy, especially using molecularly targeted drugs [9]. Gefitinib is a molecularly targeted drug that inhibits epidermal growth factor receptor (EGFR) tyrosine kinase. In Japan, it has been widely used since 2002. With the advent of gefitinib, the median OS of patients with NSCLC harboring EGFR tyrosine kinase inhibitors (EGFR-TKIs) sensitizing mutation has dramatically improved to 20 months [10, 11]. Indeed, third-generation EGFR-TKIs have become the first-line therapy for patients harboring EGFR mutation [12].

The mainstream treatment for painful bone metastasis from lung cancer is currently radiotherapy (RT), and in our experience, re-calcification of the bone destruction from metastasis is not commonly seen. However, among those lung cancer patients treated with EGFR-TKIs, many have shown a distinctive response in osteosclerotic change after bone destruction due to metastasis. Several literature studies have reported such changes [13–15]. Nevertheless, the incidence and extent of such re-ossification after using gefitinib remains unclear. Therefore, we performed a retrospective observational study to clarify the effect of gefitinib on the radiological response of patients with NSCLC harboring EGFR mutation.

## Methods

**Fig. 1:**
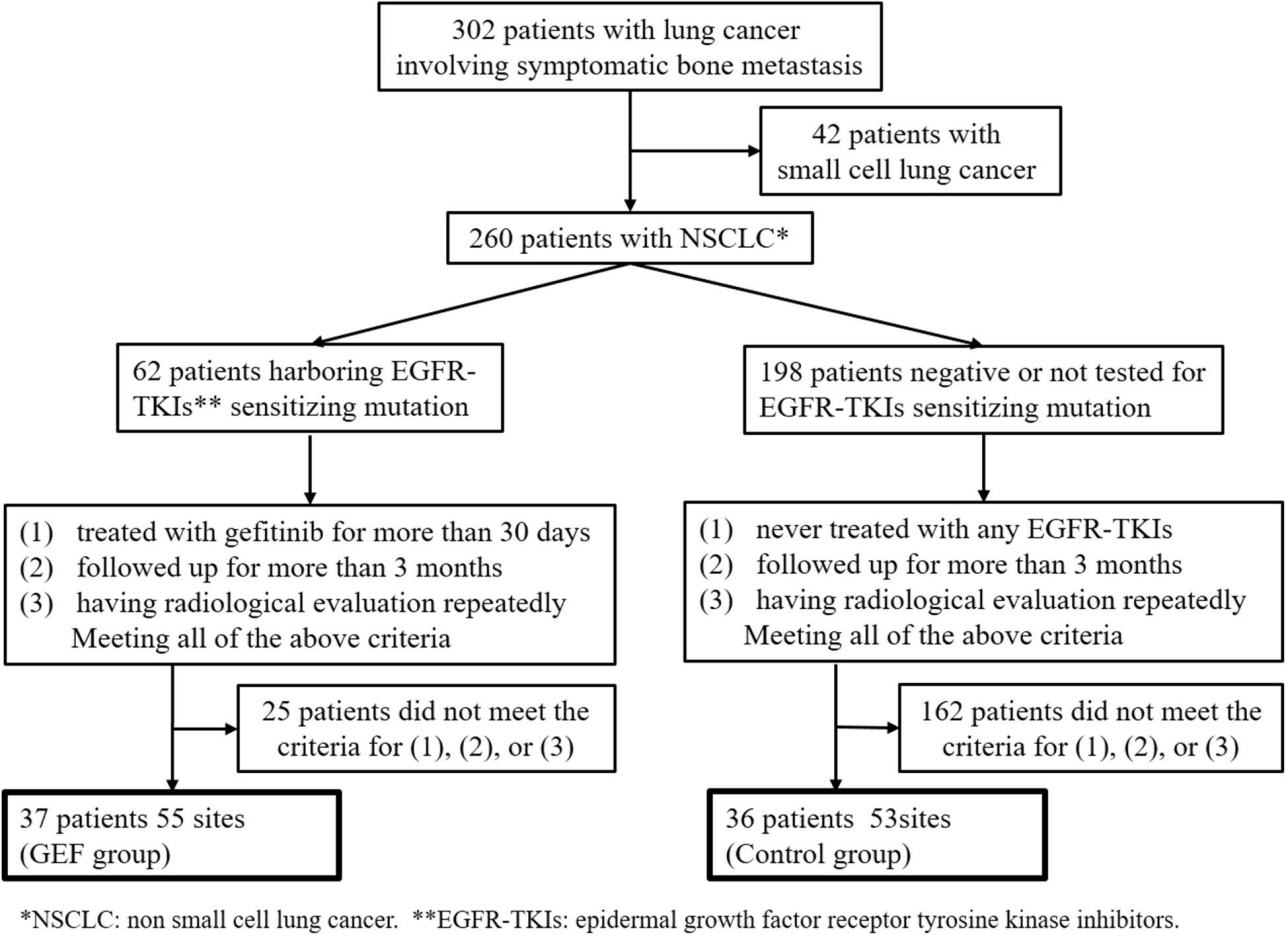
Flow chart for patients screening

Between January 2008 and December 2011, we treated 302 patients with lung cancer involving symptomatic bone metastasis. Of these 302 patients, 260 had NSCLC, of which 62 harbored EGFR-TKIs sensitizing mutation. The subjects of this study were 37 of the 62 patients with EGFR mutation that were treated with gefitinib for more than 30 days and followed up for more than 3 months with repeated radiological evaluation (GEF group). Meanwhile, of the 198 EGFR-TKIs sensitizing mutation negative or no EGFR test patients, 36 had never received EGFR-TKIs treatment and met the other follow-up and assessment criteria for inclusion. As a result, the control group comprised 53 sites in 36 patients (Fig. 1). Regarding RT for bone metastases, 39 lesions (71%) in the GEF group also received RT with the median radiation dose of 30 Gy to palliate pain (GEF with RT group) and 16 lesions did not receive RT (GEF w/o RT group). In the control group, 31 lesions (58%) received RT with the median radiation dose of 30 Gy (control with RT group) and 22 lesions (42%) did not receive RT (control w/o RT group) (Table 1).

**Table 1:** Characteristics of the patients in the study population

		GEF	Control
**Sex**	Male	14(38%)	28(78%)
	Female	23(62%)	8(22%) ^*^
**Age(year)**	Median	63	62
	Range	44–77	37–84 ^**^
**Histrogy**	Adeno	36(97%)	26(72%)
	Squamouse	1(3%)	7(19%)
	other	0(0%)	3(8%)
**EGFR mutation**	L858R	18(49%)	-
	19Del	18(49%)	-
	19DEL/L858R	1(2%)	
**Metastatic type**	Osteolytic	31(56%)	30(57%)
	Mixed	24(44%)	21(40%)
	Undetectable	0(0%)	2(4%)
**Evaluated sites**	Spine	38(69%)	35(66%)
	Pelvis	8(15%)	6(11%)
	Femur	3(5%)	3(6%)
	Other	6(11%)	9(17%)
**Gefitinib (Days)**	Median	250	-
	Range	37–763	-
**Radiotherapy**	+	39(71%)	31(58%)
	-	16(29%)	22(42%)
**1-year overall survival**		80.0%	35.3% ^***^

^*^P < 0.001

^**^P = 0.087

^***^P < 0.001

We retrospectively analyzed the radiological best overall response, the interval between the start of treatment and the appearance of radiological response, and the time to lose sclerotic response on plain X-ray or Computed tomography (CT) imaging which was calculated using local response. For the GEF group, the treatment start date was defined as the date gefitinib administration was started, and for the control group, it was defined as the day starting the first chemotherapy or RT after the diagnosis of bone metastasis. In addition, we investigated the relationship between RT and these radiological responses.

### Radiological evaluation

**Fig. 2:**
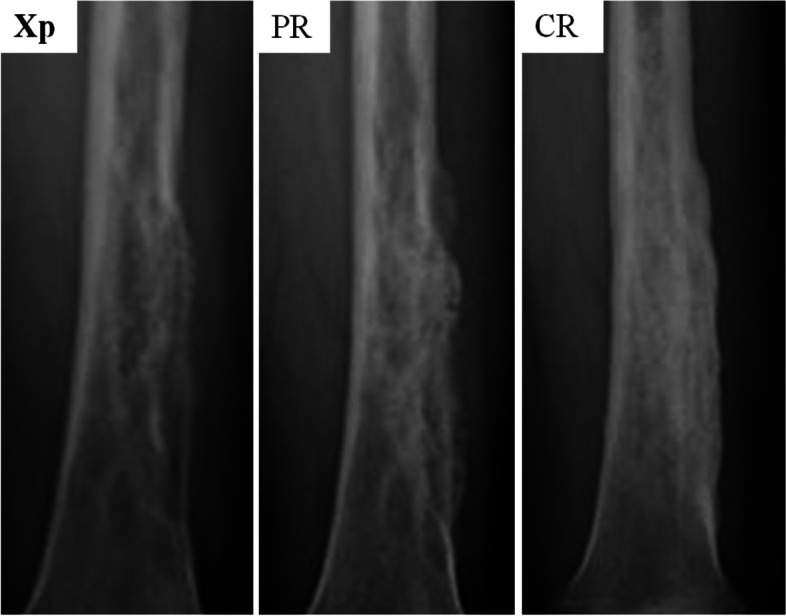
Representative radiographs showing evaluation criteria. Radiographs obtained before treatment show lytic metastasis. After some treatment, the radiographs showed “partial filling in,” categorized as partial response (PR), and “complete filling in,” categorized as complete response (CR)

**Fig. 3:**
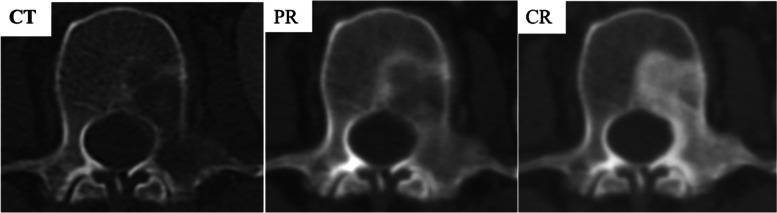
Representative computed tomography (CT) scans demonstrate evaluation criteria. CT scans obtained before treatment shows lytic metastasis. After some treatment, the scans showed “partial filling in,” categorized as partial response (PR), and “complete filling in,” categorized as complete response (CR)

For the appendicular bone, responses were evaluated on plain radiographs, and for axial bone metastasis, CT was used for evaluation. Evaluations were based on Harada’s criteria [16] for radiological evaluation. This evaluation criterion is a modification of Hamaoka’s evaluation criteria [17] developed to evaluate local response after RT. The responses were divided into four categories: complete response (CR), partial response (PR), no change (NC), and progression disease (PD; Table 2). Representative images are shown in Figs. 2 and 3.

**Table 2:** Radiological response criteria (H HARADA et al.: J. Radiat. Res. (2010))

Response Type	Initial plain radiography pattern			
	Sclerotic	Mixed	Lytic	Undetectable
Complete response (CR)	Normalization	Normalization Complete filling in	Normalization Complete filling in	Complete Sclerosis
Partial response (PR)	Regression in size	Sclerotic rim Partial filling in Regression in size	Sclerotic rim Partial filling in Regression in size	Sclerotic rim Partial filling in
No change (NC)	No change	No change	No change	No change
Progressive disease (PD)	Increase in size	Increase in size	Increase in size	Appearance of lytic lesion

### Statistical analysis

To evaluate the synergistic effects of RT in combination with gefitinib, we compared patients who received gefitinib with RT (GEF with RT group) and those treated with gefitinib without RT (GEF w/o RT group). To analyze the effect of gefitinib alone, we compared the GEF w/o RT group and the control w/o RT group. The Fisher exact method was employed to determine the difference in response rates between these groups.

To determine the time to lose osteosclerotic response, the local response maintenance rate was calculated using Kaplan–Meier analysis, wherein death with maintaining radiological response was defined as censor. The log-rank test was also used for comparison. The Mann–Whitney U test was performed to determine the difference in the average interval from gefitinib initiation to the appearance of bone response. Two-tailed 5% significance was used for all comparisons.

All statistical analyses were performed using StatView software (version 5.0; SAS Campus Drive, Cary, North Carolina, USA).

## Results

### Patients’ characteristics

Of the 37 patients in the GEF group, 18 had a point mutation in exon 21 (21-L858R), 18 had exon 19 deletion (19-del), and one had both 21-L858R and 19-del. By contrast, of the 36 patients in the control group, 22 patients had wild-type EGFR and 14 patients had no EGFR test.

Twenty-five of 37 cases in the GEF group and 19 of 36 cases in the control group had symptomatic multiple bone metastases. Among patients with multiple bone metastases, 35 patients had two separate evaluable axial bone lesions. Of these 35 patients, both evaluable lesions were included in the study. Hence, analyses were performed for 55 lesions in 37 cases in the GEF group and 53 lesions in 36 cases in the control group.

There were 14 male and 23 female patients, with a median age of 63 years (range: 44–77), in the GEF group. Gefitinib was administered for a median duration of 250 days (range: 37–763). Analyses were performed for 38 lesions in the spine, eight in the pelvis, three in the femur, and six in other locations. Meanwhile, the control group had 28 male and eight female patients, with a median age of 62 years (range: 37–84). Thirty-five lesions in the spine, six in the pelvis, three in the femur, and nine in other locations were analyzed.

The 1-year OS rates in the GEF and control groups were 80% and 35.3%, respectively (*p* < 0.001; log-rank test, Table 1). Details of patients’ characteristics are listed in Table 1.

### Comparison of best overall response between the GEF group and the control group

Radiological CR was achieved in 28 of 55 lesions (51%), radiological PR was achieved in 26 lesions (47%), and only one lesion (2%) was evaluated to have NC in the GEF group. Pleasingly, the overall response rate (CR + PR) was as high as 98% (54 out of 55 lesions), with all showing the characteristic osteosclerotic change (Fig. 2 and 3). By contrast, in the control group, three of 53 lesions (6%) showed radiological CR and 27 lesions (51%) showed radiological PR, but 10 lesions (19%) had NC and 13 lesions (25%) showed PD. The overall response rate was 57% (30 out of 53 lesions), showing a significant difference (*p* < 0.001; Fisher exact method; Table 3).

**Table 3:** The best overall response (overall response rate)

Group		Response				
		CR	PR	NC	PD	Total
GEF	RT( +)	23 (59%)	16 (41%)	0	0	39
	RT(‐)	5 (31%)	10 (63%)	1 (6%)	0	16
	total	28 (51%)	26 (47%)	1 (2%)	0	55
Control	RT( +)	3 (10%)	21 (68%)	6 (19%)	1 (3%)	31
	RT(‐)	0	6 (27%)	4 (18%)	12 (55%)	22
	total	3 (6%)	27 (51%)	10 (19%)	13 (25%)	53

**Fig. 4:**
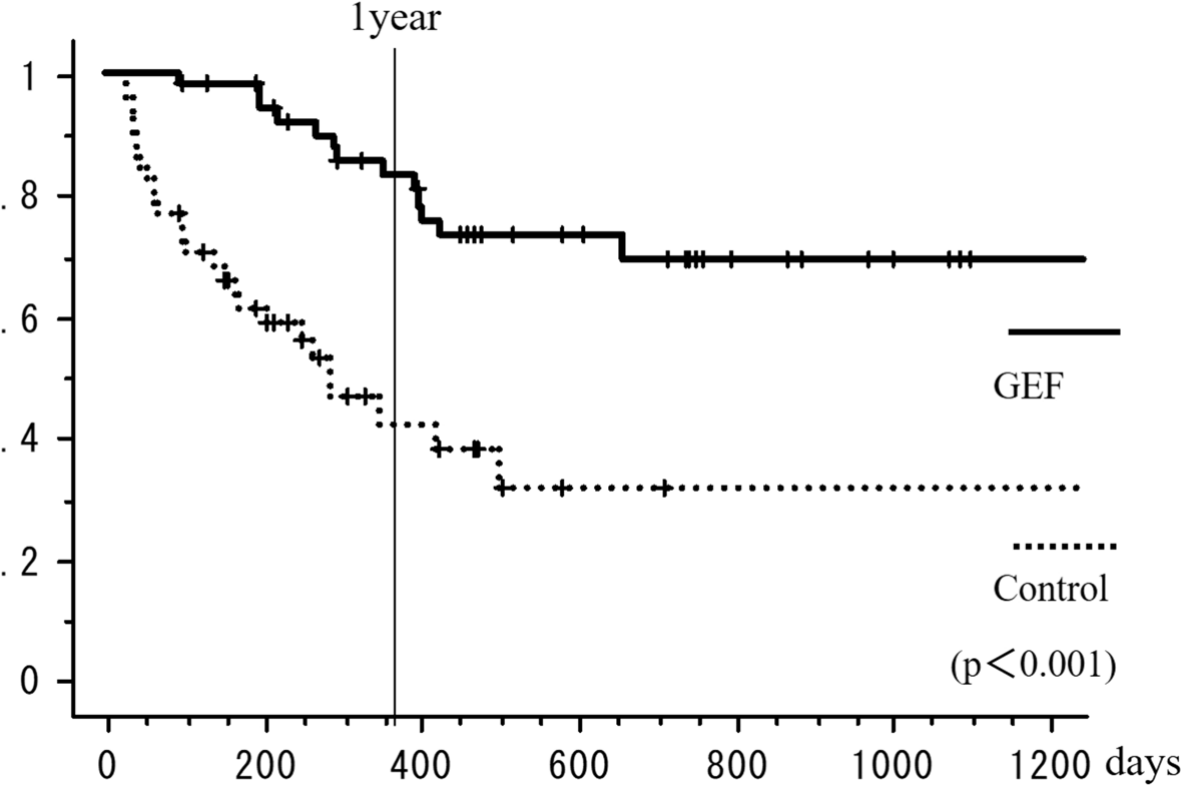
The local response maintenance rate of the GEF group and control group. The local response maintenance rate at 1 year was 83% and 42%, respectively (log-rank test *p* < 0.001)

The local response maintenance rate at 1 year was 83% in the GEF group compared to only 42% in the control group. Thus, a favorable response was maintained significantly longer in the GEF group than in the control group (*p* < 0.001; log-rank test, Fig. 4).

### Synergistic effects of Gefitinib and RT

In the GEF group, 39 lesions (71%) also received RT to alleviate pain. To assess the synergy between gefitinib and RT, we compared the GEF with the RT group and the GEF w/o the RT group. In the GEF with RT group, twenty-three of 39 lesions (59%) achieved radiological CR, and 16 lesions (41%) achieved radiological PR. The overall response rate was 100%. By contrast, in the GEF w/o RT group, five of 16 lesions (31%) achieved radiological CR and 10 lesions (63%) achieved radiological PR, and one lesion (6%) was NC. The overall response rate was 93% (15 out of 16 lesions) and showed no significant differences (*P* = 0.08; Fisher exact method; Table 3).

The average interval from gefitinib initiation to the appearance of bone response was shorter (45 days) in the GEF with RT group than in the GEF w/o RT group (57 days). However, the difference between these two groups was not significant (*P* = 0.95; Mann–Whitney U test).

**Fig. 5:**
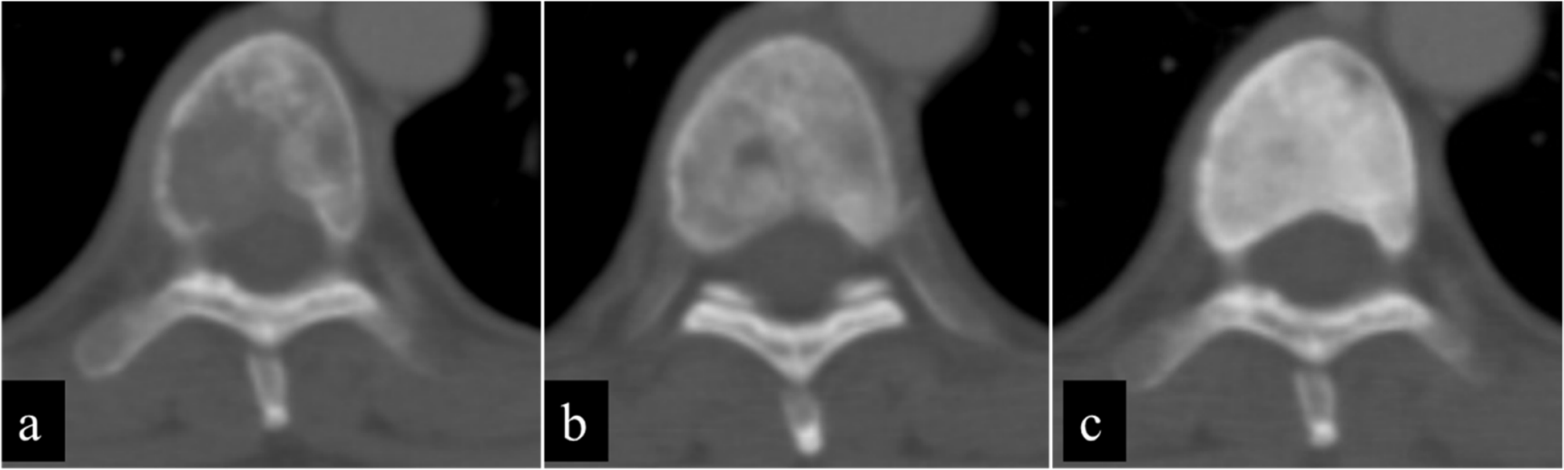
The CT scan revealed osteolytic metastasis (GEF with RT group) in the 7^th^ thoracic spine (a). To alleviate pain RT was performed first, followed by gefitinib administration for 344 days after the completion of RT. Forty-six days after starting gefitinib therapy, PR was observed on CT images (b); subsequently, CR was achieved at seven months (c). Local CR was maintained for two years and two months until her death

**Fig. 6:**
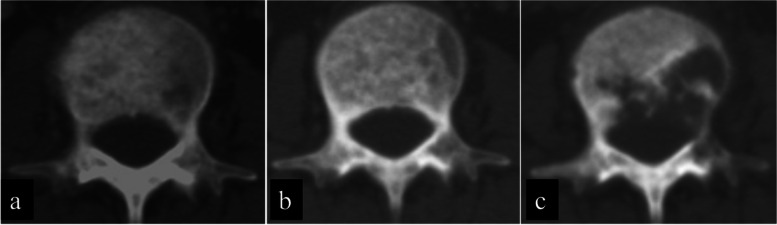
The CT scan demonstrated mixed-type 3rd lumber spine metastasis (GEF w/o RT group) (a). The patient was treated with gefitinib alone because the stability of the spine was deemed sufficient to preclude RT. Local PR was achieved as noted on CT two months after the start of gefitinib (b), However, osteolytic bone destruction became evident again after 1.2 years from the start of treatment and was considered to have lost sclerotic response (c)

**Fig. 7:**
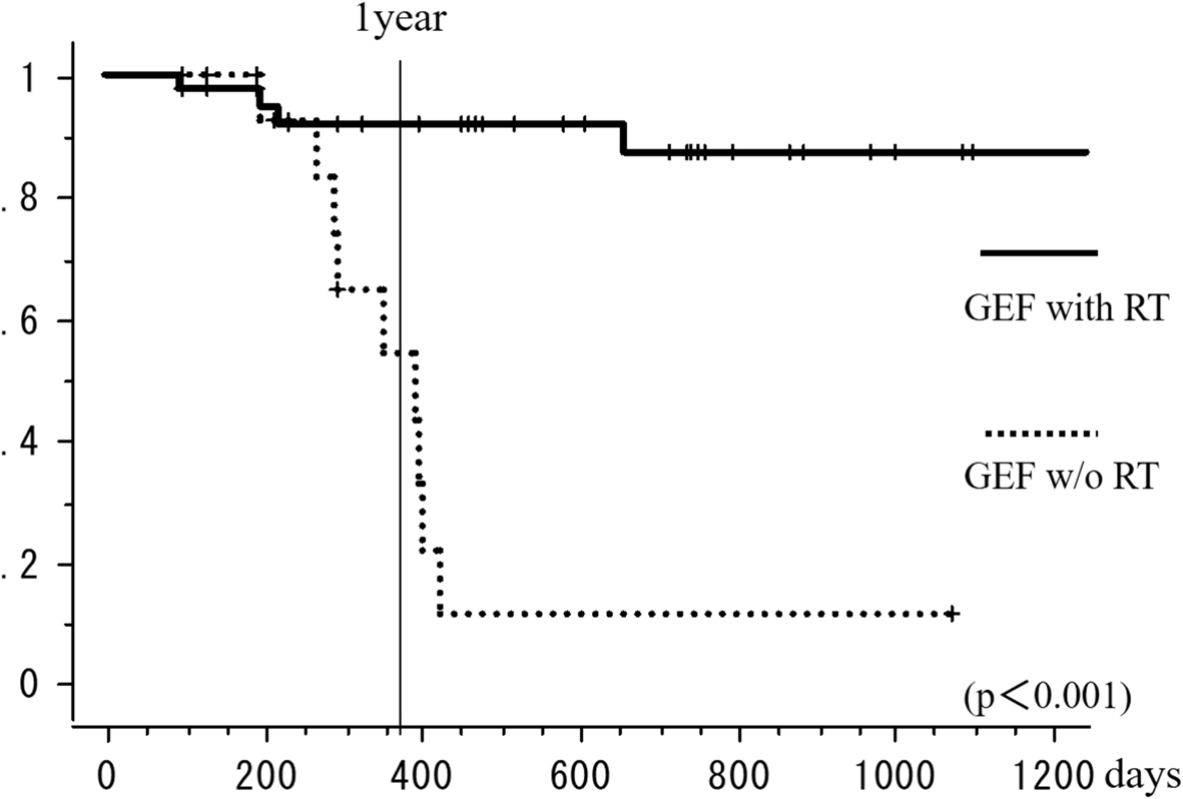
The local response maintenance rate of GEF with RT group and GEF w/o RT group. The local response maintenance rate at 1 year was 92% and 54%, respectively (log-rank test *p* < 0.001)

The loss of sclerotic response was found in 4 (10%) of 39 sites in the GEF with RT group and 9 (56%) of 16 sites in the GEF w/o RT group. The 1-year local response maintenance rate was 92% in the GEF with the RT group and 54% in the GEF w/o the RT group. Figure 5 shows a representative case of the GEF with the RT group in which sclerotic response was maintained. Figure 6 demonstrates a typical case of GEF w/o RT group in which osteolytic change re-appeared. The sclerotic response was maintained significantly longer in the GEF with RT group than in the GEF w/o RT group (*p* < 0.001; log-rank test, Fig. 7).

### Effect of gefitinib excluding the effect of RT

**Fig. 8:**
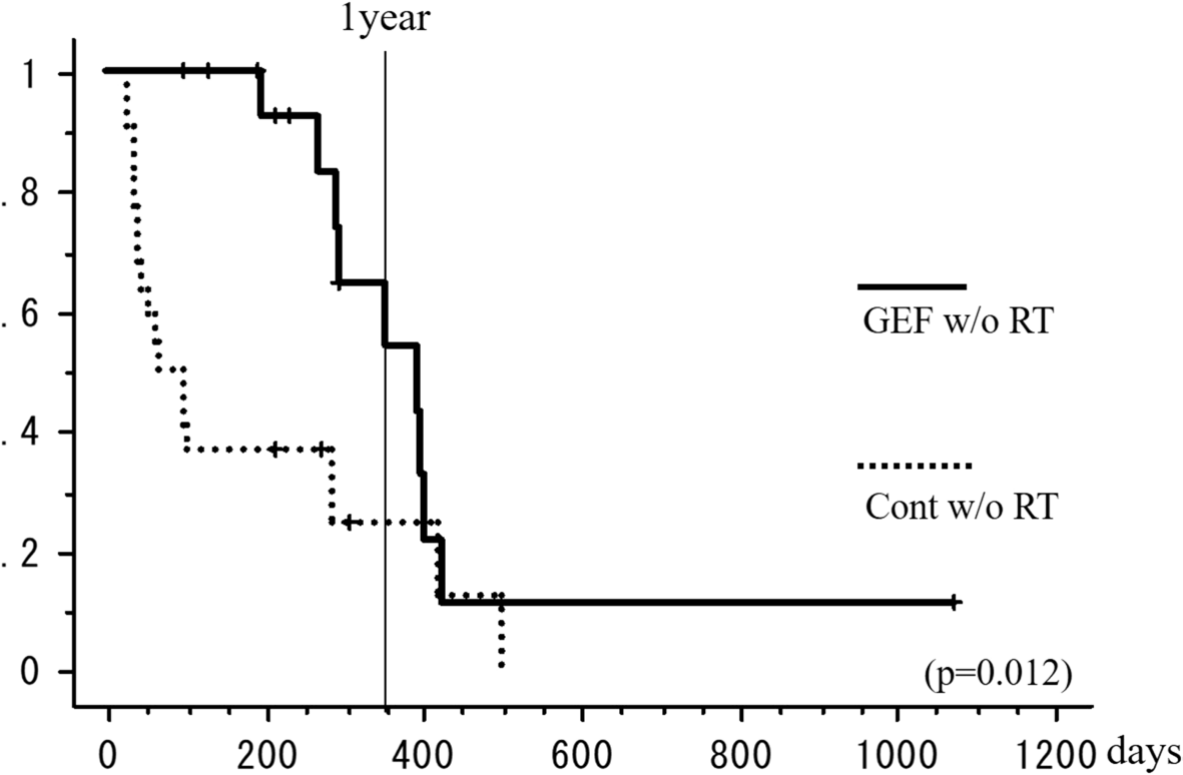
The local response maintenance rate of GEF w/o RT group and control w/o RT group. The local response maintenance rate at 1 year was 92% and 54%, respectively (log-rank test *p* = 0.012). The median local response maintenance time in the GEF w/o RT group was 11.7 months

We compared the GEF w/o RT (16 sites) and control w/o RT (22 sites) groups. The best overall response in the GEF w/o RT group was CR in five patients (31%), PR in 10 (63%), and NC in one (6%), as shown in Table 3. By contrast, the best overall response in the control w/o RT group was PR in 6 patients (27%), NC in 4 patients (18%), PD in 12 patients (55%), and there was no CR case. Consequently, the overall response rate was 94% in the GEF w/o RT group and 27% in the control w/o RT group, and there was a significant difference between these groups (*p* < 0.001; Fisher exact method, Table 3). The local response maintenance rate shown in Fig. 8 demonstrates that the responses could be maintained until 270 days (9 months) with the treatment of GEF only (GEF w/o RT group) but deteriorated after that and showed no difference with the group treated only with cytotoxic chemotherapy (control w/o RT group) (*p* = 0.019; log-rank test, Fig. 8). The median local response maintenance time in the GEF w/o RT group was 11.7 months.

## Discussion

### Radiological osteosclerotic change by gefitinib

EGFR-TKIs significantly extend progression-free survival (PFS) and median OS in patients with NSCLC harboring EGFR-TKIs sensitizing mutation [12]. However, few original studies have assessed their clinical effect on skeletal metastasis. This study is the first report to investigate the radiological effect of bone metastasis in patients with NSCLC harboring EGFR-TKIs sensitizing mutation treated with gefitinib.

The effect of conventional chemotherapy with cytotoxic drugs on bone metastases is unclear. Sun et al. reported that the skeletal-related events occurrence rate by conventional cytotoxic chemotherapy was as high as 59 out of 81 (73%). However, this skeletal-related events occurrence rate significantly decreased after EGFR-TKI treatment [18].

The present study revealed that as much as 98% of the bone metastases in patients with NSCLC harboring EGFR mutation after treatment with gefitinib responded to show osteosclerotic change, and 51% of patients achieved CR. In 2012, Yamashita et al. reported that 11 out of 41 NSCLC patients treated with gefitinib, demonstrated osteosclerotic changes [19]. Their report had excluded patients who underwent RT, other anti-cancer drugs, or bone-modifying agents but crucially, had not accounted for the presence of EGFR mutations. The osteosclerotic rate in Yamashita’s report was 27%, lower than the 98% observed in the current study. The discrepancy may well be attributable to the GEF group in the current study including only patients with EGFR mutation and not excluding patients who underwent local RT or bone-modifying agents. Approximately 30% of the patients with lung adenocarcinoma in Eastern Asia are EGFR mutation-positive [20]. Assuming that 30% of the 41 subjects in the report by Yamashita et al. had been patients harboring EGFR-TKIs sensitizing mutation, the 11 cases (27%) of osteosclerotic change could have occurred mostly in patients harboring EGFR mutation.

One might raise the question of whether the phenomenon of osteosclerosis is a predictive indicator of the prognosis. On comparing the 32 sites in the control group that were PR and CR with the 21 sites that were SD and PD, a significant difference was obtained (p < 0.001; log-rank test) with 1-year survival rates of 53.1% and 6.0%, respectively, from the initiation of the treatment. This suggested that osteosclerotic changes could be used as prognostic indicators as these may indicate the efficacy of anticancer drug therapy or radiotherapy against the lesion.

### Effects of adding RT for patients who are candidates for gefitinib

In 2010, Harada et al. reported that 42% of the patients with bone metastasis responded to RT and 10% showed CR by their criteria [16]. As we mentioned before, the current study demonstrated that radiological response in bone metastases from patients with NSCLC harboring EGFR-TKIs sensitizing mutation to gefitinib treatment without RT was PR or better in 94% and CR in 31% of cases. This result raises the question as to whether or not RT is necessary. According to our study, the GEF with RT group showed a significantly better local response maintenance rate at 1 year than the GEF w/o RT group (log-rank test *p* < 0.001; Fig. 7). The interval to show response on images also tended to be shorter in the GEF with RT group. These results indicate that combining RT with gefitinib treatment is effective to maintain local control and that it may contribute to an earlier response.

Regarding PFS of first-generation EGFR-TKIs for patients harboring EGFR mutation, it was reported to be between 9.2 and 12 months [21]; however, there are no reports about how long the sclerotic response of bone metastases continues. As shown in Fig. 8, without RT (GEF w/o RT group), the local response maintenance rate declines sharply around 300 days from the start of gefitinib therapy. Consequently, the local response maintenance rate of the GEF w/o RT group becomes similarly low to that of the control w/o RT group. This means that the sclerotic response in bone metastasis induced by gefitinib lasted for a similar period as that in the primary lesion or its visceral metastasis.

Since the present cases include a patient who received irradiation, we were interested in assessing whether bone sclerosis was achieved due to EGFR-TKI or irradiation. Among patients treated with gefitinib without RT, CR and PR rates were 94% and 78% in the control with RT group (Table 3). Closer examination suggested that 31% of the patients in the GEF w/o RT group achieved CR, while only 10% of those in the control with RT group achieved CR. These results suggested that osteosclerotic changes were predominantly brought about by gefitinib.

In this study, the osteosclerotic effect appeared as early as an average of 45 days. Osteosclerotic effects were also observed in patients who discontinued the drug due to side effects at 37 days. Therefore, even short-term administration may cause symptomatic bone metastases. However, sclerosis disappeared soon after transforming to PD, indicating that bone metastases worsened soon after EGFR-TKI was discontinued. In this regard, we believe that irradiation should be used in combination with EGFR-TKI for critical areas such as the spine or acetabulum.

### Mechanism of osteosclerotic change by gefitinib

Normanno et al. found that gefitinib inhibited induction of osteoclast differentiation through a reduction in receptor activator of nuclear factor kappa-B ligand (RANKL) expression [22]. It has been reported that EGFR mutation is involved in the RANKL pathway [23, 24] and EGF signals are involved in bone metastasis of many cancers [25, 26].

Kishimoto et al. reported an NSCLC case in which bone metastasis showed a remarkable osteosclerotic change after multimodal therapy including gefitinib [27]. Pathological specimens of the bone lesion collected at autopsy revealed trabeculae composed of cartilage matrix, woven bone, and osteoids surrounded by osteoblasts, findings associated with ossification, suggesting repair after microfracture due to weakening by the osteolytic tumor. These studies suggest that gefitinib may act in a suppressive manner on osteoclasts and promote osteosclerotic change of bone metastasis.

Currently, third-generation EGFR-TKIs are widely used. In the FLAURA trial conducted in 2017 [28], gefitinib (first generation) and osimertinib (third generation) were compared. The study demonstrated that PFS with osimertinib was 18.9 months, significantly longer than that with gefitinib (10.2 months). However, there was no significant difference in the response rate between them. In this study, we investigated patients treated with first-generation EGFR TKIs and demonstrated that the median local response maintenance rate in the GEF w/o RT group was 11.7 months, which is similar to the results of the gefitinib in the FLAURA trial. Therefore, considering third-generation EGFR-TKIs was associated with a longer PFS than first-generation EGFR-TKIs in the FLAURA trial, a longer duration of response after treatment with third-generation EGFR-TKIs for bone metastases may be expected.

The present study has limitations. First, most of the patients were diagnosed with bone metastasis based on radiological images and did not have pathological evidence. However, patients with double cancers or conditions atypical to lung cancer were excluded from this study. In addition, CT or PET-CT images were compatible with typical bone metastasis findings. Therefore, we believe that the diagnosis was correct. If multiple bone lesions show typical features of bone metastasis radiographically in advanced lung cancer, then it would be reasonable and beneficial for a patient to start treatment for lung cancer with bone metastasis without performing a bone biopsy.

Second, we only enrolled symptomatic patients with bone metastases who could be followed up for three months or more and who could be regularly followed up with imaging study; therefore, there can be a bias that drop-outs or patients who died within three months, as well as those who opted for best supportive care, were not enrolled in this study. Asymptomatic patients with bone metastases were not included in the study, partly because of the lack of frequent imaging evaluation. A prospective randomized study enrolling more patients would be necessary to establish the effect of EGFR-TKI treatment on bone metastasis conclusively.

Third, we could not exclude the effect of bone-modifying agents. Quattrocchi et al. analyzed 23 patients with bone metastasis including six cases of lung cancer and reported that an increase of at least 50% in bone density was observed in 87% of patients after three months of zoledronic acid therapy [29]. Therefore, the osteosclerotic effect in the gefitinib group may partly be enhanced by bone-modifying agents. In the current study, 56% of patients in the gefitinib group and 47% of them in the control group regularly received zoledronate.

Fourth, this study was conducted exclusively among Japanese patients. Gefitinib was first approved in Japan in 2002, followed by Europe and the U.S. in 2009 and 2015, respectively. Gefitinib is indicated for the treatment of patients positive for EGFR-TKI sensitizing mutations. However, Asians have a higher positive percentage for EGFR mutations, especially in NSCLC (38.8%), as compared to Caucasians (17.4%) and African-Americans (17.2%) [30]. Therefore, they are less likely to benefit from EGFR-TKI as compared to Asians.

Fifth, the sex ratio between GEF and the control groups varied widely. This is because, among Asians, EGFR mutation-positive lung cancers are overwhelmingly high in females [9, 30]. EGFR-TKI was indicated for patients with positive status for EGFR mutations, and thus, the GEF group comprised a large proportion of females. The number of patients in the control group was also small, making it difficult to match the ratio of male to female patients.

In summary, our study revealed that gefitinib treatment for patients with NSCLC harboring EGFR-TKIs sensitizing mutation causes osteosclerotic changes of lytic or mixed bone metastasis in 98% of patients. Moreover, the effect of gefitinib could continue up to a median duration of 355 days, which is similar to the progression-free period of gefitinib for internal organs reported in the literature. Radiotherapy for bone metastasis is effective in maintaining the osteosclerotic effect of gefitinib.

## Conclusion

Gefitinib treatment for bone metastases in patients harboring EGFR mutations resulted in a beneficial osteosclerotic change in 98% of these patients. Conventionally, the treatment of lung cancer bone metastases is RT or surgery. However, EGFR-TKI may result in bone sclerosis and improve bone fragility in patients harboring EGFR mutations. Thus, in some cases, depending on the site, EGFR-TKI alone without RT may be sufficient. Since the combination of gefitinib and radiotherapy resulted in early osteosclerosis and long-lasting local control of bone metastases, we reasonably speculate that this is the best treatment for symptomatic patients with osteolytic bone metastasis at critical sites such as the thoracic spine, acetabulum, and femur and harboring EGFR mutations.

## Data Availability

All data generated or analyzed during this study were obtained from clinical records of participating patients and were included in a database after anonymization. Patients’ image data should not be shared because they may identify individuals. The datasets used and/or analyzed during the current study are available from the corresponding author on reasonable request.

## Abbreviations

NSCLC: Non-small cell lung cancer
EGFR: Epidermal growth factor receptor
EGFR-TKI: EGFR tyrosine kinase inhibitor
OS: Overall survival
RT: Radiotherapy
CR: Complete response
PR: Partial response
NC: No change
PD: Progression disease
CT: Computed tomography
PFS: Progression-free survival
